# Optimizing Parkinson’s disease diagnosis: the role of a dual nuclear imaging algorithm

**DOI:** 10.1038/s41531-018-0041-9

**Published:** 2018-02-23

**Authors:** J. William Langston, Jesse C. Wiley, Michele Tagliati

**Affiliations:** 1 Parkinson’s Institute, 675 Almanor Ave, Sunnyvale, CA 94085 USA; 2 Department of Comparative Medicine, University of Washington, 1959 NE Pacific Ave Seattle, Seattle, WA USA; 3 Department of Neurology, Cedar-Sinai Medical Center, 127 S San Vicente Blvd, AHSP 6600, Los Angeles, CA 90272 USA

## Abstract

The diagnosis of Parkinson’s disease (PD) currently relies almost exclusively on the clinical judgment of an experienced neurologist, ideally a specialist in movement disorders. However, such clinical diagnosis is often incorrect in a large percentage of patients, particularly in the early stages of the disease. A commercially available, objective and quantitative marker of nigrostriatal neurodegeneration was recently provided by 123-iodine ^123^I-ioflupane SPECT imaging, which is however unable to differentiate PD from a variety of other parkinsonian syndromes associated with striatal dopamine deficiency. There is evidence to support an algorithm utilizing a dual neuroimaging strategy combining ^123^I-ioflupane SPECT and the noradrenergic receptor ligand ^123^I-metaiodobenzylguanidine (MIBG), which assesses the post-ganglion peripheral autonomic nervous system. Evolving concepts regarding the synucleinopathy affecting the central and peripheral autonomic nervous systems as part of a multisystem disease are reviewed to sustain such strategy. Data are presented to show how MIBG deficits are a common feature of multisystem Lewy body disease and can be used as a unique feature to distinguish PD from atypical parkinsonisms. We propose that the combination of cardiac (MIBG) and cerebral ^123^I-ioflupane SPECT could satisfy one of the most significant unmet needs of current PD diagnosis and management, namely the early and accurate diagnosis of patients with typical Lewy body PD. Exemplary case scenarios will be described, highlighting how dual neuroimaging strategy can maximize diagnostic accuracy for patient care, clinical trials, pre-symptomatic PD screening, and special cases provided by specific genetic mutations associated with PD.

## Introduction

Parkinson’s disease (PD) is the second most common neurodegenerative disorder of aging with a prevalence of about 1–2% in adults over age 60 in industrialized countries.^1^ In excess of one million people suffer from PD in the United States alone and, due to the progressive aging of the population, the importance of PD as a public health issue is expected to increase. The incidence of the disease is increasing over time, particularly in males, and it is projected that by 2030 the number of individuals with PD in the world will triple.^2^ In the United States, annual medical costs associated with PD, including doctor visits, medications, physical and speech therapies, and comorbidities of the disease (such as depression and dementia) are significant burdens to the healthcare system, with a total cost to the nation projected to be $23 billion annually.^3^

The burden of disease is likely even greater considering the widespread presence of Lewy bodies in PD^4^ and the multifaceted symptoms and signs associated with this syndrome.^5,6^ Put another way, our traditional definition of parkinsonism is just the “tip of the iceberg” of what PD truly appears to be.^7^ And if Lewy bodies remain a cardinal pathologic feature of PD, it is now clear that PD is just one aspect of a larger synucleinopathy, best described as multisystem Lewy body disease (MLBD) and comprising PD, pure autonomic failure (PAF), and dementia with Lewy bodies (DLB).^8^

These evolving concepts regarding the widespread nature of PD synucleinopathy has also led to challenges in diagnosis, which, as typically defined, relies on the clinical acumen of an experienced neurologist, ideally one who is specialized in movement disorders. For decades, diagnosis has been based on the recognition of four cardinal signs: resting tremor, bradykinesia, rigidity and postural instability. However, recent evidence has shown that the clinical diagnosis is often incorrect in an alarming percentage of patients, particularly in the early stages of the disease.^9^ Obviously, this could affect day-to-day patient care and seriously confound the interpretation of clinical trials. In this paper, we provide supportive evidence for a dual imaging algorithm that could markedly improve the diagnostic accuracy of MLBD, including its dominant clinical manifestation—idiopathic PD.

## The advent of ^123^I-ioflupane SPECT

123-iodine ^123^I-ioflupane SPECT imaging (also known by its trade name DaTscan^TM^) provides a commercially available, objective and quantitative marker of nigrostriatal neurodegeneration. Radiologists, nuclear medicine physicians and neurologists can directly visualize the loss of dopamine transporters in the striatum of PD patients, the loss of which increases in severity with disease progression. ^123^I-ioflupane SPECT abnormalities can detect dopaminergic deficiency at the threshold of motor pathology, as early as Hoehn & Yahr (H&Y) Stage I.^10^ Despite its widespread use and recent FDA approval in the US, the diagnostic role of ^123^I-ioflupane SPECT is limited by a number of issues, including: (1) the difficulty to differentiate PD from a variety of other parkinsonian syndromes associated with striatal dopamine deficiency; (2) an inferior spatial resolution as compared to other imaging techniques, particularly positron emission tomography (PET); (3) the possibility of false negatives in subjects with clinical evidence of parkinsonism, also known as “scans without evidence of dopaminergic deficits” (SWEDD); (4) the possibility that dopaminergic treatment impacts the imaging techniques; (5) how subtle a dopaminergic deficit can be detected, potentially limiting its utility in addressing early-stage transitions from pre-motor PD to symptomatic PD; and (6) its economic cost and safety profile in terms of exposure to radiations.

The major diagnostic limitation of ^123^I-ioflupane-SPECT is the inability to clearly differentiate PD from other parkinsonian syndromes associated with striatal dopamine deficiency, including multiple systems atrophy (MSA), progressive supranuclear palsy (PSP), and corticobasal degeneration (CBD).^11^ For this reason, the FDA approval notes the use of DaTscan for distinguishing between parkinsonian syndromes and ET, but not between idiopathic PD (iPD) and atypical parkinsonian patients.^2^

Although SPECT spatial resolution near 1 cm (8–12 mm) may be less accurate than other nuclear imaging approaches like PET,^12^ ^123^I-Ioflupane-SPECT, and ^18^F-labeled dopamine precursor (^18^F-DOPA) PET have generally shown to be comparable in their ability to detect changes in presynaptic dopaminergic activity in the striatum.^12,13^ PET studies investigating other DAT binding ligands, such as ^11^C-PE2I, demonstrate comparable ability to detect DAT deficits, although the ^11^C-PE2I-PET can simultaneously assess cerebral perfusion.^14^ However, the short half-life of ^11^C-labeled compounds makes this approach unfeasible for all but large facilities with a cyclotron locally available.^12^ ^123^I-labeled versions of PE2I have also been reported to have lower binding to the serotonin transporter SERT, likely due to PE2I’s 10-fold lower SERT affinity;^15^ although the clinical significance is still undetermined, preliminary studies suggest that ^123^I-Ioflupane-SPECT binding to both DAT and SERT may have limited use in differential diagnosis between some parkinsonian syndromes.^16^

Another limitation of ^123^I-Ioflupane-SPECT is the characterization of subjects that clinically appear to have parkinsonism, but do not have decreased striatal DAT signal, also known as SWEDD. The idea that these cases may represent some form of early-stage parkinsonism that will convert to DAT negative PD over time is not supported by long-term longitudinal studies.^17,18^ These individuals remain a hot topic of investigation, particularly within the ongoing longitudinal Parkinson’s progression marker initiative (PPMI).

Whether dopaminergic treatment impacts ^123^I-Ioflupane-SPECT imaging is a matter of controversy. Ioflupane is a cocaine derivative and drugs of abuse acting on the dopaminergic system (i.e., methamphetamine and cocaine) impact ^123^I-Ioflupane-SPECT binding to DAT in the striatum^19^ to the point that the European Association of Nuclear Medicine Neuroimaging Committee (ENC) recommends ceasing these drugs prior to imaging. However, imaging data support little to no impact of levodopa and dopamine agonists on striatal ^123^I-Ioflupane-SPECT binding.^20^ As levodopa is converted to dopamine by amino acid decarboxylase (AADC) within the synaptic terminal, it is not expected to compete with the imaging agent in the synaptic cleft^19^. Nevertheless, other drugs commonly used to treat schizophrenia and depression can impact ^123^I-Ioflupane-SPECT binding,^19,21^ and careful consideration must be made before deciding how to handle comorbid patients.

Finally, ^123^I-Ioflupane-SPECT costs are not trivial, running up to 2500–3000 dollars depending on local prices and institutions in the US, and may provide further limitations to the widespread use of the technology. Exposure to radiation is within FDA safety ranges and hydration can minimize radiation doses to the bladder during the excretion phase of the DaTscan.

## The rationale for a dual imaging algorithm

There is evidence to support an algorithm utilizing a dual neuroimaging strategy, which combines the ability of ^123^I-ioflupane SPECT to assess the integrity of the nigrostriatal dopaminergic system and the noradrenergic receptor ligand ^123^I-metaiodobenzylguanidine (MIBG) (also known as Adreview^TM^) to assess the post-ganglion peripheral autonomic nervous system. Before explaining the rationale behind this algorithm, it is important to review the evolving concepts regarding the synucleinopathy that affects the central and peripheral autonomic nervous systems as part of a multisystem disease that includes PD, DLB, and PAF.^8^

### The central nervous system

The neuropathology of PD has a lengthy history, dating back to the early 1900s.^22^ Interestingly, F.H. Lewy first identified the inclusion bodies that now bear his name in the locus coeruleus, nucleus basalis of Meynert, and the dorsal motor nucleus of the vagus nerve,^22^ but not the substantia nigra—where they were first described by Tretiakoff in 1919.^15^ It was not until the 1950s that Greenfield called attention to the importance of substantia nigra in PD^23^. The subsequent focus on the nigrostriatal dopaminergic system has dominated modern PD research for at least 50 years, although we now know that damage to the substantia nigra represents only one aspect of the pathology.^24^ In fact, it has become increasingly apparent that there is consistent “geographical” evolution of the disease in the brain. Using histological techniques based on the discovery that Lewy bodies and neurites are strongly stained by antibodies to alpha-synuclein, Braak et al.^25^ developed a new staging system suggesting that PD first appears in the brainstem (dorsal motor nucleus of the vagus nerve, Braak Stage I), then spreads rostrally over time to the pons and mesencephalon, causing cell loss in the locus ceruleus (Stage II) and substantia nigra (Stage III), until it eventually involves the basal forebrain and the cerebral cortex (Stage VI). It is worth noting that nearly 20 years before Braak’s staging, Kosaka et al.^26^ hypothesized a similar progression, dividing the stages into brainstem, transitional, and diffuse. In one aspect, this system is more intuitive in that it clearly implies that at the latest stage the disease is widespread and does not involve only the cortex, as the term “dementia with Lewy bodies” implies.

PD notably affects two other CNS structures: the olfactory bulb, which is affected very early in the disease (Braak Stage I), and the spinal cord. The significance of the consistent presence of Lewy body pathology in the olfactory bulb is unknown.^27^ Further, it does not fit either of the staging systems described above, leading some to surmised that the olfactory bulb could be an entry portal for an exogenous toxin or infectious agent.^28^ The spinal cord can be affected as well, but typically only after the detection of Lewy pathology in the brain, which suggests that sporadic PD does not begin in the spinal cord, but travels into the spinal cord concomitant with progression of Lewy pathology in the brain.^22,29^

### The peripheral autonomic nervous system

*The key to the diagnostic algorithm*: The effects of PD outside the CNS were largely unappreciated until recently. However, a careful review of the literature reveals that the evidence has been there for nearly 80 years. Herzog first described Lewy bodies in the superior cervical sympathetic ganglia in 1937,^30^ and the disease has been known to widely affect the peripheral autonomic nervous system for over 60 years.^31^ In the 1980s, the identification of Lewy bodies in the mesenteric plexus of the GI tract provided further evidence for a role of the autonomic system.^32^ Indeed, a widespread autonomic neuropathy characterized by synuclein-positive Lewy bodies and Lewy neurites is now generally considered an integral part of PD.^33^

### Getting to the heart of the matter

In 1990, Goldstein and colleagues reported that sympathetic denervation of the heart could be imaged using the noradrenergic PET ligand [18F]-6-fluorodopamine, providing the first evidence that the autonomic neuropathy associated with PD affected the heart.^34^ Since that time, over 40 studies using adrenergic receptor ligands have shown that cardiac sympathetic denervation is a “near universal” feature of PD.^35^ Most of these studies have used noradrenaline analog MIBG, which is taken up, stored and released in sympathetic nerve terminals. Radioactively labeled with either [^123^I] or [^131^I],MIBG provides a surrogate marker of sympathetic nerve integrity and activity. The cardiac sympathetic deficits identified by MIBG appear to represent the same pathological process that is occurring in the brain, as synuclein-based Lewy bodies and Lewy neurites, as well as TH loss have now been documented in the heart.^36^ Indeed, one recent autopsy study unambiguously identified synuclein-positive Lewy bodies and neurites in the cardiac tissue of 100% of the examined PD patients.^37^ Finally, MIBG was recently validated for the first time in a clinicopathologic study, in which cardiacMIBG uptake, in the early and delayed phases, was respectively reduced in 90.9% and 95.7% of patients who were later proven pathologically to have Lewy body disease.^38^

MIBG technology shares some of the limitations previous listed for ^123^I-Ioflupane-SPECT, and in particular low spatial resolution, as compared to PET scan, and cost. PET scan has been equally established to study the cardiac autonomic nervous system and provides a number of advantages over SPECT technology, including high spatial and temporal resolution, in addition to a variety of radiolabeled catecholamines, catecholamine analogs, and receptor ligands considered more physiologic than MIBG.^39^ The use of SPECT/CT technique may improve diagnostic MIBG accuracy.^40^ In addition, methodological demands and costs make PET technology less widely available and virtually limited to research centers.

### Other Lewy body diseases—dementia with Lewy bodies (DLB) and pure autonomic failure (PAF)

If DLB and PAF are all part of a multisystem Lewy body disorder, replete with a widespread peripheral autonomic nervous system involvement, they should also have cardiac sympathetic denervation including the heart.^8^ Thus, one would expect cardiacMIBG imaging to be abnormal in both these disorders, and indeed this is typically the case. Abnormal MIBG scans have been well documented in patients with DLB,^41,42^ to the point that it is now being used to differentiate DLB from Alzheimer’s disease (AD),^43^ which is not surprising as AD lacks a peripheral autonomic neuropathy. Finally, completing the triad of MLBD, a decrease in MIBG uptake has been reported in PAF,^44^ another condition shown to be predominantly associated with Lewy body pathology.^45^ In summary, it is now well established that MIBG deficits are a common feature of MLBD (PD, DLB, and PAF).^35^ In view of this, it seems reasonable to suggest that MIBG can be used as a unique and unifying feature these three synucleinopathies.

### Pre-motor symptoms

Strong evidence links idiopathic REM-behavior disorder (iRBD) and MLBD in patients over the age of 50. Although it is not clear if every patient with iRBD will ultimately develop parkinsonism or dementia, over 80% of subjects in Schenk’s original iRBD cohort have developed symptoms of the MLBD complex (parkinsonism or dementia) 16 years after entering the study.^46^ When additional prodromal synucleinopathy markers are also considered, the risk of developing MLBD is even higher. For example, individuals with iRBD and abnormal olfaction have an 80% risk of developing neurodegeneration after 8 years.^47^ The neurodegenerative risk associated with iRBD is not surprising as the locus coeruleus, a structure thought to a part of RBD pathophysiology, is typically affected in the second stage of the Braak progression, before the involvement of the substantia nigra.^22^ The strong association between a diagnosis of RBD and the detection of a Lewy body synucleinopathy at autopsy further supports this hypothesis.^48^ MIBG uptake has been extensively studied in individuals with iRBD and RBD associated with PD,^49–51^ suggesting that MIBG deficits may be detected in subjects with Lewy body pathology before the onset of classical parkinsonian signs.^52,53^ Interestingly, MIBG deficits also appear to correlate with hyposmia in early-stage PD.^54^ Although hyposmia is not unique to PD, the correlation of MIBG with the olfactory impairment further suggests that MIBG may be a potent marker of pre-motor PD,^54,55^ and that cardiac sympathetic denervation is an integral part of the widespread synucleinopathy of PD. MIBG defects have been described also in patients with constipation, another well described pre-motor symptoms of PD.^53^ In summary, MIBG offers a remarkable opportunity to detect a synucleinopathy prior to the onset of motor deficits and/or cognitive issues with a high degree of certainty, at a time when the damage caused by neurodegeneration is presumably less, giving disease modification efforts a much better chance to succeed.

### Homing in on typical Lewy body PD

As noted above, ^123^I-ioflupane SPECT allows for the documentation of nigrostriatal system damage, but cannot be used to sort out PD from atypical forms of parkinsonism.^11^ What does distinguish atypical parkinsonism from all forms of MLBD, including PD, is the lack of peripheral post-ganglionic autonomic neuropathy. Therefore, cardiacMIBG imaging should be highly effective in differentiating Lewy body PD (as well as DLB and PAF) from other neurodegenerative forms of parkinsonism and dementia, which mimic PD in the early stages of progression. In view of this, it should not be surprising that numerous studies have shown that MIBG heart to mediastinum ratio (HMR) is far lower in PD patients than in those with atypical parkinsonism, including MSA, PSP, and CBD.^56,57^ Several systematic reviews and meta-analyses have addressed the capacity of MIBG to detect PD and to differentiate it from other atypical parkinsonian syndromes. One of these carefully examined the primary data from 12 previous studies and found that MIBG consistently differentiates PD from MSA, PSP, and CBD.^58^ Despite considerable variation across individual studies, the combined values yielded a sensitivity of 89.7% and a specificity of 82.6%, demonstrating an unprecedented capacity to isolate PD from other atypical form of parkinsonism.^58^ This meta-analysis went on to examine a subset of studies that presented data on early PD cases (Hoehn & Yahr Stage I or II) and compared the accuracy of MIBG in differentiating this patient subpopulation from MSA, PSP and CBD patients. The resulting pooled sensitivity was 94.1% with a specificity of 80.2% at what is arguably the most difficult diagnostic time point in the course of PD.^58^ Breakdown comparisons were made isolating results in PD and MSA (sensitivity: 90.2, specificity: 81.9) as well as in PD and PSP (sensitivity: 91.4, specificity: 78). These values are closely aligned with those reported in other systematic reviews of the literature.^59^ Overall, these studies provide a compelling body of evidence that MIBG imaging can distinguish between classic idiopathic Lewy body PD and other parkinsonian syndromes. The sub-group analysis differentiating early-stage PD patients from those with MSA is consistent with other original reports examining MIBG efficacy in detecting early stages of PD.^60,61^

Although there are isolated reports of MIBG deficits in MSA patients, these studies show quantitatively less of an impact of MSA on the HMR than observed in PD patients.^62,63^ There is disagreement about the stage of disease showing the greatest MIBG diagnostic value in distinguishing parkinsonian syndromes, with some data pointing to the early stages of progression,^64^ whereas others find that greater divergence occurs late in the disease.^65,66^ Overall, the preponderance of studies demonstrate a lack of MIBG abnormalities in MSA^57,64,67–69^ or PSP,^56,65^ whereas PD patients have values consistently and significantly lower than controls, MSA, PSP, or CBD patients.^62,65,70–73^ The fact that none of the MIBG studies in MSA patients involved autopsy confirmation may be a key to resolving most, if not all, of these conflicting reports. Recently, a consortium including our group reported pathological findings in 133 patients who carried a diagnosis of MSA until the time of their death.^74^ Surprisingly, the diagnosis of MSA was correct in only 62% of cases, with Lewy body disease accounting for the majority of misdiagnosed patients. Thus, misdiagnosis of MSA likely explains the variable results in these ante-mortem studies, particularly as a significant number of such cases appear to actually have a Lewy body disease. Indeed, one could make the case that any report on MSA without autopsy confirmation may have been confounded by inclusion of a significant number of patients with other parkinsonian disorders, including PD.

### MIBG, ^123^I-ioflupane, and dual imaging algorithm

Several studies have compared the predictive power of dopaminergic degeneration as imaged by either ^123^I-ioflupane SPECT with MIBG imaging. Both techniques appear to be effective at identifying PD patients with similar sensitivity and specificity,^60,75^ and both MIBG^76,77^ and ^123^I-ioflupane SPECT binding^78^ appear to decrease over time and have the potential to be used as viable biomarkers of disease progression. However, ^123^I-ioflupane SPECT imaging provides specific mechanistic information on the stage of degeneration within the dopaminergic axis that MIBG cannot provide. ^123^I-ioflupane SPECT analysis also correlates better with the degree of motor impairment,^60^ although ^123^I-MIBG uptake has been found to be lower in patients with akinetic-rigid parkinsonism or postural instability gait disorder.^79^ On the other hand, as this review has emphasized, MIBG can differentiate PD from other parkinsonian syndromes,^75,80^ whereas ^123^I-ioflupane SPECT cannot. Furthermore, MIBG abnormalities have been associated with typical pre-motor symptoms such as hyposmia,^54^ REM-behavior disorder (RBD),^81^ and constipation,^82^ raising the possibility that MIBG may be more effective at detecting the earliest stages of MLBD, even at the pre-motor stages, and specific non-motor subtypes or routes of pathology spread.^79^ These two imaging techniques have already been used in combination^80,83^ as a tool to select patients for deep brain stimulation^84^ and to sort out patients with mixed tremors^85^ and “de novo” PD patients from other forms of parkinsonism.^60^ Of note, MIBG is now included as supportive criteria in the revised MDS clinical diagnostic criteria for PD.^86^

**Fig. 1:**
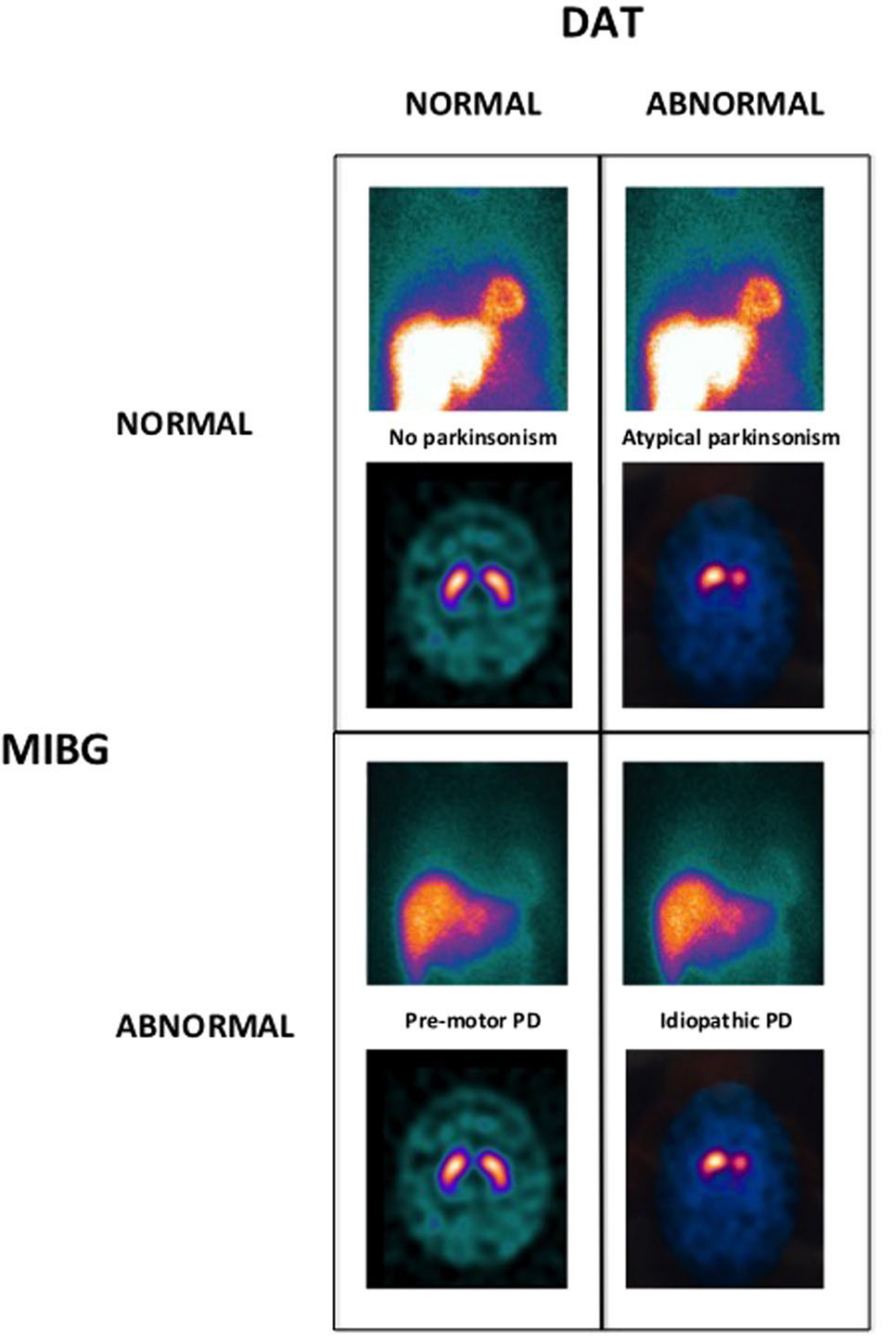
MIBG-DAT dual imaging diagnostic algorithm. MIBG abnormality separates multisystem Lewy body disease (MLBD) pathologies, including idiopathic Parkinson’s disease (PD) and dementia with Lewy bodies (DLB) from other types of parkinsonism. ^123^I-Ioflupane measures the integrity of nigrostriatal dopamine projections. Low dopamine transporter (DAT) levels in the caudate, putamen or both, in either brain hemisphere, are consistent with a parkinsonian disorder, independently of its nature. In subjects with appropriate symptoms and signs, the combination of the two imaging modalities allows discriminating four distinct pathologies: (1) non-parkinsonian disorders (upper left quadrant, dual test normality); (2) atypical non-Lewy body parkinsonism (upper right quadrant, normal MIBG, and abnormal DAT); (3) early-stage MLBD or pre-symptomatic parkinsonism (lower left quadrant, abnormal MIBG and normal DAT); (4) MLBD, either DLB or PD (lower right quadrant, abnormal MIBG, and DAT)

## Areas of impact of the MIBG/^123^I-ioflupane algorithm

On the basis of the evidence summarized above, the combination of cardiac (MIBG) and cerebral ^123^I-ioflupane SPECT could satisfy one of the most significant unmet needs of current PD diagnosis and management, namely the early and accurate diagnosis of patients with typical Lewy body PD.^80,87,88^ Large-scale validation studies of these combined techniques should ideally comprise a sufficient number of subjects in four subgroups of study subjects including patients with idiopathic PD, subject with highly predictive pre-motor symptoms (RBD, hyposmia, and/or other criteria), patients with atypical parkinsonism (MSA or PSP), and normal subjects or patients with essential tremor, as a control group (Fig. 1). The following case scenarios highlight how combined MIBG and ^123^I-ioflupane SPECT imaging can be combined to maximize their diagnostic capacities.

### Patient care

Needless to say, accurate and early diagnosis has major implications for patient management, both in terms of appropriate treatment and prognosis. In case of abnormal ^123^I-ioflupane SPECT abnormality, an MIBG scan can be used to separate Lewy body disease (PD, DLB, PAF) from other forms of neurodegenerative parkinsonism,^40,44,89^ which can mimic Lewy body PD, particularly in early stages of the disease. In addition, MIBG may prove useful to characterize cases with a clinical diagnosis of PD and ^123^I-ioflupane SPECT scans lacking evidence of dopaminergic defects (SWEDD). A recent study showed that subjects with SWEDD do not have the same level of MIBG deficit that is observed in PD, supporting the suggestion that they may have an alternative diagnosis, prognosis, and pathway for clinical management.^90^

### Clinical trials

The natural first step in developing new and effective therapeutics for PD is the accurate diagnosis of the candidate clinical population. The difficulty associated with this task is compounded by the relative complexity of early-stage diagnoses. Numerous studies have shown that there is a tendency within the neurological community to over-diagnose PD,^91^ and a recent clinic-pathologic study by Adler and colleagues^9^ found that, within the first five years, a diagnosis of “probable PD” was incorrect 50% of the time. In patients diagnosed with “possible PD” the error rate rose to a remarkable 74% during the first 5 years. In these settings, using the ^123^I-ioflupane SPECT /MIBG algorithm would allow for the selection of more accurately diagnosed patients, thus greatly enhancing the power of clinical trials. This is a critical point, as the development of effective therapeutics depends on a homogenous clinical population with a precisely defined pathology. Furthermore, clinical trials with disease modifying therapies typically seek to intervene in early stages of PD, making the accurate selection of an intent-to-treat clinical population much more challenging. This diagnostic difficulty involves two fundamental problems, which can be addressed with a dual imaging algorithm, including: (1) the differentiation of parkinsonism from similar neurological syndromes such as essential tremor (the use for which the ^123^I-ioflupane SPECT is FDA approved) and dystonia,^85^ and (2) the accurate distinction of idiopathic PD from other neurodegenerative forms of parkinsonism that have vastly divergent disease etiology, pathology and sensitivity to treatment,^89^ as well as, drug-induced parkinsonism that can result from treatment with antipsychotic medications.^92^

Given the large body of clinical and pathological evidence, there is reason to predict that the dual imaging algorithm may accurately identify appropriate clinical populations for therapeutic trials for Lewy body PD vs other parkinsonian syndromes. More specifically, eligibility for PD therapeutic clinical trials ideally should require both an abnormal ^123^I-ioflupane SPECT and a diminished MIBG HMR, whereas participation in clinical trials focused on other parkinsonian syndromes (MSA, PSP, and CBD) will be determined by an abnormal ^123^I-ioflupane SPECT in the presence of a normal MIBG scan (Fig. 1). MRI imaging can be used to further separate the atypical form of parkinsonism in a substantial number of cases.^93^

### Special cases: LRRK2 G2019S mutation carriers

The LRRK2 G2019S mutation is the most common genetic abnormality associated with Lewy body parkinsonism.^94^ However, a recent study from a consortium that includes our institute found that only two-thirds of patients with LRRK2 G2019S-associated parkinsonism have classic Lewy body PD at autopsy.^95^ Up to 30% showed focal brainstem degeneration, affecting particularly the substantia nigra and the locus coeruleus; interestingly, this alternative LRRK2 G2019S pathology appears to have a tau-based molecular etiology. Intriguingly, the results of our pathology study are in line with small earlier study demonstrating that only 3 of 6 parkinsonian patients carrying the G2019S LRRK2 mutation had an abnormal MIBG scan,^96^ suggesting that the mutation may lead to different pathological phenotypes with divergent treatment needs and prognosis. It can be argued that the dual imaging algorithm with ^123^I-ioflupane SPECT and MIBG would be the best approach to separating these phenotypes in life. Indeed, the ^123^I-ioflupane SPECT and MIBG-SPECT algorithm is being increasingly used to sort out the genetic forms of parkinsonism, as over 30 genetic variants have been reported to cause PD, but only a few appear to have MLBD-based pathology and/or MIBG scans.^8^ In short, the dual imaging algorithm could become very important for the study and treatment of the genetic parkinsonisms to identify which genes cause MLBD disease and which do not.

### Pre-symptomatic PD screening

The dual imaging algorithm using MIBG and ^123^I-ioflupane SPECT scans may fulfill one of the largest unmet needs for PD, which is the ability to confirm the presence of pre-motor and pre-dementia MLBD when screening procedures suggest it may be present. Although there is abundant evidence that cardiac denervation occurs well before the onset of parkinsonism,^52,81^ it would be impractical and cost ineffective to use nuclear medicine scans as a tool to screen the general population. However, the population at risk can be narrowed with other markers of sympathetic cardiac denervation, such as reduced heart rate variability (HRV)—as measured by R–R intervals over time using a simple 5-min EKG. We have shown that this technique can disclose HRV abnormalities in RBD patients similar to those seen in PD patients.^97^ Of note, HRV often decreases as well in DLB patients,^38,42,98^ which is also a part of MLBD. Routine EKGs and self-administered smell tests could be woven into annual physical examinations of individuals after 50 years as a way to screen the general population for evidence of pre-motor PD. Patients with an abnormal HRV and smell tests (and/or other pre-motor symptoms or signs) would then be candidates for undergoing the dual imaging algorithm. Those in which results point to an early synucleinopathy would become candidates for disease modifying therapies, if and when such therapies become available. Finally, this approach has the potential to pre-symptomatically separate PD patients from atypical forms of parkinsonism, and DLB patients from other forms of dementia including Alzheimer’s disease.^83,99,100^

## Conclusion

A large body of literature and experimental evidence supports the notion that the combined utilization of MIBG and ^123^I-ioflupane SPECT offers a powerful biomarker for MLBD and could have profound impact on the diagnosis and management of PD, offering both more accurate diagnosis and early points of intervention that are critical to therapeutic development and clinical trials, as well as effective patient care. This approach is gaining a sense of urgency given the unacceptable frequency of misdiagnosis based on clinical examination alone, particularly during the first 5 years of the illness.^9^ The dual ^123^I-ioflupane SPECT and MIBG scan algorithm appears to be able to address this urgent need.
